# Natural killer cells in COVID-19: from infection, to vaccination and therapy

**DOI:** 10.2217/fvl-2022-0040

**Published:** 2023-03-14

**Authors:** Alireza Zafarani, Mohammad Hossein Razizadeh, Salar Pashangzadeh, Mohammad Reza Amirzargar, Mahsa Taghavi-Farahabadi, Mohammad Mahmoudi

**Affiliations:** ^1^Department of Hematology & Blood Banking, School of Allied Medicine, Iran University of Medical Sciences, Tehran, Iran; ^2^Department of Virology, Faculty of Medicine, Iran University of Medical Sciences, Tehran, Iran; ^3^Iranian Research Center for HIV/AIDS, Iranian Institute for Reduction of High-Risk Behaviors, Tehran University of Medical Sciences, Tehran, Iran; ^4^Immunology Today, Universal Scientific Education & Research Network (USERN), Tehran, Iran; ^5^Department of Immunology, School of Medicine, Tehran University of Medical Sciences, Tehran, Iran; ^6^Department of Immunology, School of Medicine, Iran University of Medical Sciences, Tehran, 1449614535, Iran

**Keywords:** COVID-19, exhaustion, NK cells, SARS-CoV-2, vaccination

## Abstract

Natural killer (NK) cells are among the most important innate immunity members, which are the first cells that fight against infected cells. The function of these cells is impaired in patients with COVID-19 and they are not able to prevent the spread of the disease or destroy the infected cells. Few studies have evaluated the effects of COVID-19 vaccines on NK cells, though it has been demonstrated that DNA vaccines and BNT162b2 can affect NK cell response. In the present paper, the effects of SARS-CoV-2 on the NK cells during infection, the effect of vaccination on NK cells, and the NK cell-based therapies were reviewed.

SARS-CoV-2 belongs to the family Coronoviridae and is the causative agent of the COVID-19 [1], which has been declared a pandemic by the WHO. Previous coronaviruses that have caused epidemics in the past are the SARS and the Middle East respiratory syndrome viruses. The main difference between COVID-19 and past epidemics is the higher transmissibility of SARS-CoV-2, such that the virus can spread faster and easier through respiratory droplets [2]. The incubation period of the disease ranges from 2 to 14 days, and most infected hosts are asymptomatic but can still spread the disease [1]. Fever, fatigue, non productive coughs, diarrhea, headache, sore throat, loss of smell and taste are common symptoms of COVID-19. Patients with severe forms may experience dyspnea, chest pain and loss of mobility [3]. Viral infection increases levels of IL-1β, IL-6, IL-7, IL-8, IL-10, GM-CSF, granulocyte colony-stimulating factor, interferon-inducible protein 10 (IP10), monocyte chemoattractant protein-1 (MCP-1), macrophage inflammatory protein-1β (MIP-1β) and TNF-α. This can lead to cytokine storms in severe cases, which can lead to deadly consequences of acute respiratory distress syndrome and multiple organ failure [4,5].

Natural killer (NK) cells are lymphocytes of innate immunity and one of the most important members of the body’s antiviral defense. These cells are divided into two groups: CD56^bright^, which possess more cytokine production ability and CD56^dim^, which have more cytotoxic ability [6]. Unlike other lymphocytes, which identify their target cells through specific receptors, these cells lack a specific receptor and are activated by a balance between activating and inhibitory receptors [7]. NK cells have two classes of receptors: inhibitory and activating. The first class includes the NK group (NKG) receptors and killer immunoglobulin-like receptors (KIRs) such as KIR2DL1, KIR2DL2/3, KIR3DL1 and T-cell immunoglobulin and ITIM domain (TIGIT). The second class includes NKG2D, NKG2C, KIR2DS1, KIR2DS2/3, KIR3DS1, KIR2DS4 and DNAX accessory molecule-1 (DNAM-1) [8]. NK cells understand how to become activated according to the proportion of these receptors in a procedure called education. These cells avoid attacking host cells and tissues by recognizing the histocompatibility complexes (HLAs) of the host. However, virus-infected and cancerous cells reduce their surface HLAs to evade T cells. This procedure results in their recognition by NK cells [9]. NK cells destroy their targets in three ways: by ordering them to start apoptosis through the Fas/FasL pathway; by directing their destruction through perforin and granzyme; and by recruiting other immune cells via cytokine productions [10]. The crucial role of NK cells in the defense against viruses has been proven, and it is known that people with an impairment in the production and function of their NK cells have a greater chance of contracting viral diseases [11–13].

We reviewed the effect of SARS-CoV-2 on the count and function of NK cells during infection, the effect of vaccination on NK cells and also some NK cell-based therapies which can help to treat the disease.

## SARS-CoV-2 affects the frequency, phenotype & function of the NK cells

### Frequency

NK cells establish the frontline of defense against viral infections. It has been observed that a reduced number predisposes an individual to several diseases, such as different types of cancers and viral infections [11]. For this reason, researchers have evaluated the number of NK cells in peripheral blood (PB) and bronchoalveolar lavage (BAL) of patients diagnosed with COVID-19 (Table 1) [14–20].

**Table 1.** A summary of studies on NK cells in COVID-19.

Title	Sample	Sample Size	Results	Ref.
Frequency				
Characteristics of peripheral lymphocyte subset alteration in COVID-19 pneumonia	PB	60 P, 245 HC	NK cells in COVID-19 patients were significantly lower compared with HC. In severe cases, the number of NK cells was also lower than in patients in a mild situation	[19]
Impaired NK cell counts and cytolytic activity in patients with severe COVID-19	PB	10 P, 78 HC	Impaired NK cell counts and their cytolytic activity was observed in COVID-19 patients compared with HC. Plus, the cytokines which are important for NK cells' activity, including IL-12, IL-15 and IL-21 were not detected. Besides, the serum level of soluble CD25 which is negatively correlated with percentages of NK cells was significantly elevated	[17]
Immunologic perturbations in severe COVID-19/SARS-CoV-2 infection	PB	12 HC, 7 moderate P, 27 severe P, 6 recovered P	Wide changes in NK cells during COVID-19. NK cells returned to normal range after recovery	[14]
Unique immunological profile in patients with COVID-19	PB, BALF	32 P, 25 HC	Absolute NK cell count was significantly lower in COVID-19 patients compared with controls, whereas the NK cell frequency did not differ between the two groups	[18]
NK cell activation related to clinical outcome of COVID-19	PB	27 P, 17 HC	Adaptive NK cell expansion was observed	[16]
Single-cell landscape of bronchoalveolar immune cells in patients with COVID-19	BALF	13 P, 3 HC	The number of NK cells increased in BALF of COVID-19 patients	[15]
Heightened innate immune responses in the respiratory tract of COVID-19 patients	BALF	8 P, 146 CAP, 20 HC	The number of NK cells decreased in BALF of COVID-19 patients	[20]

Phenotype and function				
SARS-CoV-2 Spike 1 protein controls NK cell activation via the HLA-E/NKG2A pathway	PB	4 HC	Intracellular expression of SARS-CoV-2 Spike protein 1 causes lower NK cell degranulation via the HLA-E/NKG2A pathway	[21]
An inflammatory profile correlates with decreased frequency of cytotoxic cells in coronavirus disease 2019	PB	48 P, 20 HC	The number of NK cells expressing perforin decreased in patients in ICU	[22]
Transplantation of ACE2 mesenchymal stem cells improves the outcome of patients with COVID-19 pneumonia	PB	10 (7 MSCs transplant, 3 Placebo)	CXCR3 NK cells were increased significantly in comparison to HC which results in a cytokine storm. In contrast, in patients who underwent MSC transplantation, the overreacted NK cells disappeared	[23]
Identification of druggable inhibitory immune checkpoints on NK cells in COVID-19	PB	82 (10 HC, 10 paucisymptomatic COVID-19, 34 pneumonia, 28 ARDS due to COVID-19)	The expression level of NKG2C remained intact	[24]
Deletion of the NKG2C receptor encoding KLRC2 gene and HLA-E variants are risk factors for severe COVID-19	DNA from respiratory swabs	361 P	Patients with NKG2C/HLA-E variants are more susceptible to development of severe SARS-CoV-2	[25]
Unique immunological profile in patients with COVID-19	PB, BALF	32 P, 25 HC	The expression of NKG2D and DNAM-1, known as activating receptors of NK cells, was reduced in COVID-19 patients compared with HC	[18]
A single-cell atlas of the peripheral immune response in patients with severe COVID-19	PB	7 P, 6 HC	Most COVID-19 patients’ NK cells expressed exhaustion markers, including LAG3, TIM-3, PDCD1 and HAVCR2	[26]

Education				
NK cell activation related to clinical outcome of COVID-19	PB	27 P, 17 HC	No changes were observed in KIR receptors’ expression on NK cells	[16]
Identification of druggable inhibitory immune checkpoints on NK cells in COVID-19	PB	82 (10 HC, 10 paucisymptomatic COVID-19, 34 pneumonia, 28 ARDS due to COVID-19)	Robust expression of KIR2DL1/S1 receptors on NK cells’ surface. Besides, No changes were observed in other KIR receptors	[24]

ARDS: Acute respiratory distress syndrome; BALF: Bronchoalveolar lavage fluid; CAP: Community acquired pneumonia patients; HC: Healthy control; ICU: Intensive care unit; NK: Natural killer cell; P: Patient; PB: Peripheral blood.

**Figure 1.**
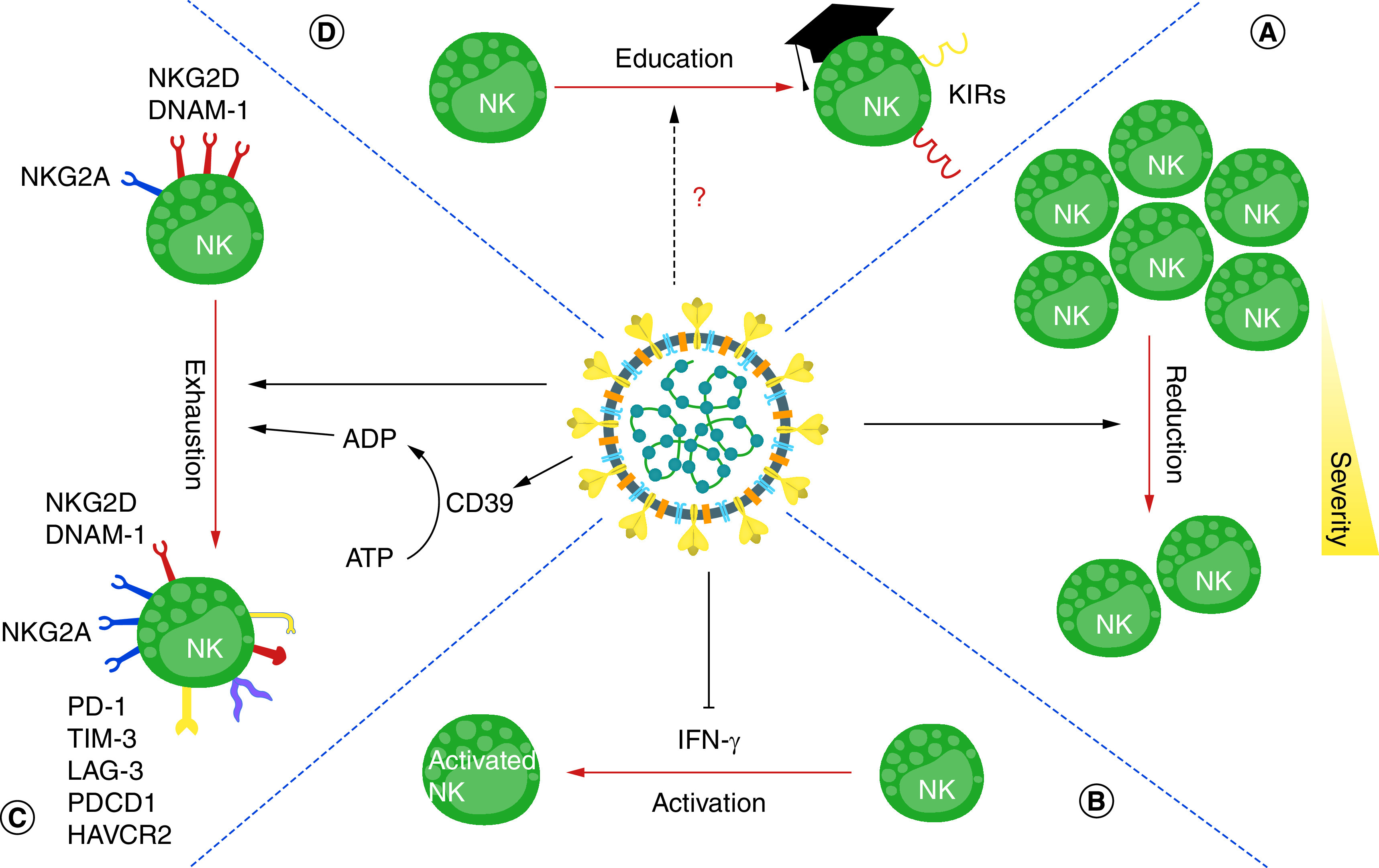
The effects of SARS-CoV-2 on the frequency, phenotype, function and education of NK cells. **(A)** SARS-CoV-2 decreases the number of natural killer cells, which is directly correlated to the severity of disease. **(B)** The production and signaling of IFN-γ are impaired. **(C)** SARS-CoV-2 infection causes the exhaustion of NK cells. CD39 is one of the enzymes that is released after the death of infected cells and plays an important role in inhibiting NK cells, by converting ATP to ADP. **(D)** SARS-CoV-2 can affect the education process of NK cells. NK: Natural killer cell; IFN: Interferons.

Like cytotoxic T lymphocytes and T helper lymphocytes, several studies have indicated that, in SARS-CoV-2 infection, the number of NK cells is decreased and is directly correlated to the severity of the disease (Figure 1A). The reduction is more noticeable in patients with severe infection than in patients with mild infection [17,19]. In these patients, among the NK cell population, the number of CD56^dim^ and CD56^bright^ NK cells is higher and lower, respectively, compared with healthy control individuals [17]. However, several studies have shown that this reduction in NK cells occurs without any differences in cell subpopulations [27,28].

In addition to disease severity, the reduction in NK cell number also depends on the stage of the disease [29]. Since NK cell numbers recover in the later stages of COVID-19, their numbers appear to decrease during the acute phase of the disease. A gradual decline in the NK cell count has been observed in patients with a severe and fatal course of COVID-19 [30]. Recently, Witkowski *et al.* showed that the number of NK cells is directly related to the amount of viral load reduction in hospitalized COVID-19 patients. Notably, faster viral clearance was observed in patients with normal NK cell counts compared with those with low NK cell counts, regardless of the clinical status [31]. Due to the increment of neutrophils in the PB, the neutrophil to lymphocyte ratio increases in patients [32,33]. The results of these studies were similar to what was observed in the SARS epidemic [34]. Studies on recovered COVID-19 patients have shown that the NK cell count does return to the normal range after recovery [14]. However, other studies have also demonstrated that the number of circulating NK cells increased significantly after the resolution of the infection [35,36]. For example, patients with long-COVID demonstrated higher levels of NK cells in the bloodstream in comparison with recovered patients [37].

The increased percentage of adaptive NK cells in the PB of COVID-19 patients has been observed [16,18]. Possible causes include: similarly to the cytomegalovirus, SARS-CoV-2 causes the production of these cells; adaptive NK cells are resistant to apoptosis after cytokine storm; or they express a particular pattern of chemokines which prevent them from leaving the blood and going to tissues [16]. However, studies on BAL showed mixed results. Liao *et al.* indicated that NK cell number in BAL increases, while Zhou *et al.* reported the reduction of NK cells. Since the sampling time in Zhou *et al.’*s study was closer to the early stages of the disease, they suggest that the trafficking of NK cells into the lungs is a time-dependent procedure that reduces the number of NK cells in the PB in the early stages of disease [15,20].

It has been observed that the expression of the vascular cell adhesion molecule 1 (VCAM-1) adhesion factor on epithelial cells is increased in patients with severe COVID-19 [38]. NK cells use the Very Late Antigen-4 to attach to VCAM-1 and leave the PB, in order to traffic to the lungs [39]. This may be another reason for the reduced number of NK cells observed in the PB.

The exact reason for the reduction of NK cells in the PB has not yet been determined. However, it is thought to be either due to the migration of NK cells from the bloodstream to the site of infection, or apoptosis [19,40]. BAL samples from COVID-19 patients were analyzed by single-cell RNA sequencing, confirming high levels of NK cells in the lungs during the acute phase of disease. This suggests that NK cells may contribute to lung tissue damage and epithelial cell death [41]. It has also been reported that NK cell-activating cytokines such as IL-12, IL-15 and IL-21 are reduced in individuals with COVID-19 [17]. Furthermore, the virus can cause apoptosis through the cytokine storm by its direct entry or indirectly [40,42]. Overall, the virus can evade the immune response and spread throughout the body by favorably altering the arrangement of immune system cells through the mechanisms described.

### Phenotype & function

Like SARS, angiotensin-converting enzyme 2 (ACE-2) is the receptor that SARS-CoV-2 uses for cell entry into host cells [43]. When the virus binds to this receptor, it activates innate immune receptors such as the toll-like receptor family, which activates signaling pathways such as NF-κB, activator protein-1 (AP-1) and the IFN-regulatory factor. These signaling pathways cause the secretion of proinflammatory cytokines such as IL-1, TNF, IL-6 and antiviral cytokines such as IFNs. To prevent the virus’s spread to other cells, these cytokines will trigger other immune cells, including neutrophils, macrophages, dendritic cells (DCs), NK cells and adaptive immune cells. In a normal immune system, these events prevent the virus from spreading, but SARS-CoV-2 has ways to bypass the immune system and use it against the host [44].

NK cells compose 10–20% of lung lymphocytes. The mature cells have the CD56^dim^ phenotype and have high lethality, but because the lung environment is anti-inflammatory, they have a high activation threshold [45]. IFNs are important human antiviral cytokines. In addition to preventing the spread of the virus by activating antiviral genes in cells adjacent to the virus infected cell, they activate NK cells promptly and suppress the virus [46]. As mentioned, with the anti-inflammatory environment of the lungs caused by the alveolar macrophages, NK cells have a high activating threshold and the timely and adequate secretion of type I IFNs is critical to activate lung NK cells [45]. SARS-CoV-2, like previous epidemic Coronaviruses, interferes with the production and signaling of these cytokines and prevents the activation of NK cells, as one of the first and most important cells acting against viral infections (Table 1) [44,47]. As the first cells are infected with the virus, other lung epithelial cells lack the activated antiviral genes to fight the virus and thus they initiate pyroptosis (a type of programmed cell death) one after another. In the event of this, large amounts of cytokines and inflammatory chemokines are released, resulting in the summoning of unnecessarily high numbers of immune cells [48,49]. In addition, it is suggested that SARS-CoV-2 can cause cell pyroptosis in lymphocytes through the activation of inflammasome [50]. One of the most critical functions of IFN-γ is to prevent neutrophils from entering the lungs and force them to activate apoptosis, but because of the impaired production and signaling of IFN-γ in COVID-19 (Figure 1B), neutrophils accumulate extensively in the lungs. This is an essential factor in the pathogenesis of the disease [51].

The CD94/NK group 2 member A (NKG2A) is an inhibitory receptor often expressed on T cells and NK cells' surface. HLA-E is the ligand for this receptor, whose expression is increased in infected cells [52]. By binding to the HLA-E, SARS-CoV-2 Spike protein-1 can reduce the NK cells' degranulation ability by the HLA-E/NKG2A pathway [21]. In NK cells, this receptor is responsible for tolerance of and preventing host cell damage [52]. This receptor is also a sign of NK cell exhaustion due to chronic infections [53]. In samples taken from patients with COVID-19, it has been observed that the level of this receptor on NK cells is significantly increased. This again is much higher in patients with severe disease [54]. Reasons for this increase include increased levels of cytokines such as IL-6 and IL-10 [55]. By reducing IL-2 and IFN-γ, IL-10 is negatively correlated with NK cell cytotoxicity. Furthermore, IL-6 reduces the expression of perforin and granzyme B [56,57].

The NKG2A receptor also plays an important role in reducing the secretion of IFNs, IL-2, TNF-α and granzyme B [55,58]. Altogether, decreased levels of IFN-γ and TNF-α along with the reduced number of NK cells and increased levels of both IL-10 and IL-6 reduce NK cells cytotoxicity [59–63]. A diminished cytotoxicity phenotype during viral infections leads to antigenic stimuli accumulation, maintenance of inflammation and tissue damage [59,62]. In another study performed by Bordoni *et al.*, functional cytotoxicity markers including perforin were reported to be lower in COVID-19 patients in the intensive care unit in comparison to non intensive care unit patients and healthy individuals [22]. Despite the decrease in NK cell subpopulations in COVID-19, Leng *et al.* found that the levels of CXCR3^+^ NK cells are increased in patients in critical conditions. This marker, which is more common among the CD56^bright^ subpopulation, might indicate that the balance of NK cells subpopulations is shifted toward an inflammatory phenotype rather than cytotoxic [23]. Due to the increase in the number of ligands of the NKG2A receptor on infected cells, and also the increased numbers of the receptor itself on NK cells, it can be concluded that SARS-CoV-2 uses NKGA2 to escape from the immune system [55].

In another study, no change was observed in the NK cell-activating receptors CD94/NKG2C in COVID-19 patients [24,64]. This receptor’s ligand is also HLA-E, but has a lower affinity than for NKG2A [65]. It can be assumed that the virus drives its spread by increasing NKG2A and its ligand, as well as keeping NKG2C constant. This causes NK cells to become exhausted during infection (Figure 1C) [24,64]. However, it has been shown that the downregulation of NKG2A in COVID-19 patients is counterbalanced by NKG2C upregulation. Vietzen *et al.* showed the deletion of the NKG2C gene and expression of the HLA-E*0101 variant on host cells were more likely to develop severe COVID-19 and be hospitalized [25]. Others have shown that NKG2C^+^ NK cells from COVID-19 patients were also characterized by a higher expression of KIRs and CD57 [27]. Herrera *et al.* showed that NKG2C+/CD57^+^ NK cells from convalescent subjects are able to secrete IFN-γ and enhance specific immune responses to soluble SARS-CoV-2 peptides [66]. In long-COVID patients, elevated levels of CD56^+^/CD57^+^/NKG2C+ NK cells are extended while having impaired virus specific and specific effector functions [37].

CD39 is one of the enzymes that is released after the death of infected cells and plays an important role in inhibiting the immune system, especially NK cells. It does this by converting ATP to ADP. Researchers have shown that the expression of CD39 is increased in the blood of patients with COVID-19. One of the reasons for this increase may be significantly increased IL-6 levels. PD1 is another inhibitory marker that is increased on the surface of NK cells in these patients. PD-L1 is the ligand of this receptor, which inhibits immune cells and causes exhaustion [24].

NKG2D is another activating receptor on the NK cell surface. This receptor’s ligands are significantly increased on virus infected cells, and include MHC class I chain-related protein A and B (MICA/B) and unique long 16 (UL16) [67]. This receptor’s expression is reduced in COVID-19 patients, which significantly weakens the immune system’s antiviral ability [18,67]. Similarly, DNAM-1 is an NK cell-activating receptor whose ligands include CD155 and CD112. This receptor is essential for the secretion of inflammatory cytokines and the cytotoxic properties of NK cells [68]. Like NKG2D, the expression of this activating receptor is reduced in patients with COVID-19 (Figure 1C) [18]. Altogether, the virus evades NK cell antiviral activity and spreads throughout the body by increasing the expression of inhibitory receptors and their ligands, as well as reducing the expression of activating receptors.

NK cells are generally known for their antiviral and anti-cancer roles, but these cells also have a strong immunomodulatory role. They play an important role in preventing damage to the host’s tissues by destroying activated immune cells such as CD8^+^ T cells and macrophages in the final stages of infection, when the threat of infection has subsided [69]. The main cause of pathogenesis in COVID-19 patients is overactivation of the immune system and the cytokine storm, which can cause severe damage to cells and tissues [70]. As mentioned, SARS-CoV-2 causes the exhaustion of NK cells by upregulating inhibitory receptors and downregulating activating receptors, thus disrupting the timely secretion and signaling of IFNs. This ultimately weakens the antiviral activity and immunomodulatory properties of NK cells [71]. In patients diagnosed with COVID-19, some exhaustion markers including lymphocyte-activation gene 3 (LAG3), T cell immunoglobulin and mucin domain containing protein 3 (TIM-3), programmed cell death protein 1 (PDCD1) and hepatitis A virus cellular receptor 2 (HAVCR2) have been characterized on NK cells (Figure 1C). However, the exact mechanisms of expression of these markers are not fully understood and further studies are required to evaluate their impact on NK cells during SARS-CoV-2 infection [26].

For controlling viral infections, the cooperation of both the innate and adaptive immune systems is required. After being infected by SARS-CoV-2, the strength and longevity of IgG are considered essential factors of immunity against COVID-19 disease. NK cells directly kill virus infected cells using different mechanisms: receptor mediated apoptosis antibody dependent cell-mediated cytotoxicity and degranulation. By interacting with DCs and secreting specific cytokines, NK cells may play an important role in the antigen presentation processes and thus explain why they are important in IgM/IgG antibody response [72–76].

Similar to the adaptive immune system, in which autoreactive T cells and B cells are eliminated, autoreactive NK cells should also be inactivated [77]. In the education process, NK cells learn how to respond to foreign agents and if an NK cell lacks tolerance to the host, it will be disabled. In this process, usually controlled by a particular class of receptors called KIRs, NK cells must have at least one inhibitory receptor of this class whose ligands are also present on host cells (e.g., KIR2DL1 and its ligand HLA-C2). Otherwise, NK cells are inactivated to avoid auto-reactivity [78]. The high number of inhibitory receptors on the surface of NK cells is associated with more efficient functioning [78]. However, the presence of ligands of NK cells activating receptors increases their auto reactivity [78]. Education is not a static process, and can be changed by the alteration of the environmental condition of the body. It is presumed that the changes in the ligands of host cells are responsible for this phenomenon. For example, in hematopoietic stem cell transplantation, the newly produced NK cells show different patterns of education [79]. Hence, surveying the possible changes in the NK cell ligands in patients with COVID-19, which can affect the education process, is important (Figure 1D).

Uneducated NK cells help the immune system by activating antigen presenting cells (APCs) via the production and release of inflammatory cytokines during inflammatory conditions [80]. The initial activation of the immune system and proper immune response to SARS-CoV-2 can restrict the infection in the initial phases, as has been observed in CMV infection [80]. Further investigation of this mechanism in SARS-CoV-2 infection is therefore required.

NK cells, as key natural effectors in the immune system, play an important role in regulating adaptive immune responses in various models of viral infection, autoimmunity and transplantation [81]. NK cells modulate T-cell responses directly through T-NK interactions by the secretion of cytokines, or indirectly through DCs and other cells. The cytolytic activity of NK cells is mediated either by direct secretion of cytolytic granules containing perforin and granzyme or by signaling through death receptors ligand pathways, such as TRAIL, NKG2D and FasL [82]. Upregulation of NKG2D on NK cells during chronic lymphocytic choriomeningitis virus (LCMV) infection and accumulation of TRAIL+ NK cells in chronic hepatitis B virus and murine cytomegalovirus (MCMV) results in the ablation of CD4^+^ and CD8^+^ T cells [83,84]. Interestingly, not only CD4^+^ and CD8^+^ T cells are eliminated by NK cells, as Treg can also be killed by NK cells [85]. NK cell secreted IL-10 is also a potent suppressor of T-cell immunity, being secreted after various infections by LCMV, MCMV, Toxoplasma gondii and Listeria monocytogenes [85–87]. Interactions between NK cells and APCs may also reduce T-cell activation. Such negative regulation, by limiting the availability of antigen presenting APCs and reducing their ability to stimulate, has been shown following infection with MCMV, LCMV and chronic human HCV, resulting in enhanced viral persistence [83,88]. Further studies are needed to focus on the negative effects of NK cells in adaptive immunity in COVID-19.

## Induction of effective immune response by COVID-19 vaccines, with involvement of NK cells

Since innate NK cells play an important role in the first line of defense against viruses, it is expected that vaccination will also affect the function or differentiation of these cells. However, few studies have focused on this issue: Watanabe *et al.* have shown that vaccination of rhesus macaques for attenuated simian immunodeficiency virus resulted in the activation of NK cells by IL-15 [89]; another study has also demonstrated that the influenza vaccine improves cytokine-induced memory-like NK cells [90]; in individuals vaccinated for influenza, it has been observed that in the vaccine responders, the population of NK cells expressing NKG2C increased after vaccination [91]. The effect of vaccination on NK cell differentiation has been evaluated in studies [92], with vaccination against Ebola and yellow fever having been shown to increase the proliferation and numbers of CD56^dim^ and CD56^bright^ subtypes [93,94].

Limited studies have evaluated the effect of COVID-19 vaccines on NK cells. In a 2020 study by Jingyou Yu *et al.*, the effects of a series of DNA vaccines on rhesus macaques were evaluated. Vaccination was shown to induce anti-Spike-dependent NK cell responses in vaccinated rhesus macaques [95]. A further study examined serological responses and predictive markers of BNT162b2 in haemato-oncological patients, and observed that the lack of serological response is associated with low levels of NK cells [96]. In a BNT162b2 mRNA vaccine trial by Cuapio *et al.*, they characterized NK cells in healthy individuals and immunocompromised patients. The numbers of NK cells, and also their subsets, phenotypes and function were assessed through consecutive PB samples at 0, 10, 21 and 35 days following vaccination, and a positive correlation was observed between the frequency of NKG2C^+^ NK cells at day 0 and anti-SARS-CoV-2 antibody titer following BNT162b2 mRNA vaccination on Day 35 [97].

There is also evidence for the effect of emerging adjuvants on NK cell populations. Adjuvant system 03 (AS03) can enhance adaptive immune responses by activating and proliferating NK cells [98]. AS01 can also increase vaccine immunogenicity by increasing the production of IFN-γ from NK cells [99]. The MF59 adjuvant can increase the efficacy of the influenza vaccine in the elderly by activating NK cells [100]. Therefore, various studies can evaluate the effect of different COVID-19 vaccines platforms or adjuvants on the proliferation, phenotype or function of NK cells.

## NK cell-based & NK cell-targeting therapies for COVID-19

**Figure 2.**
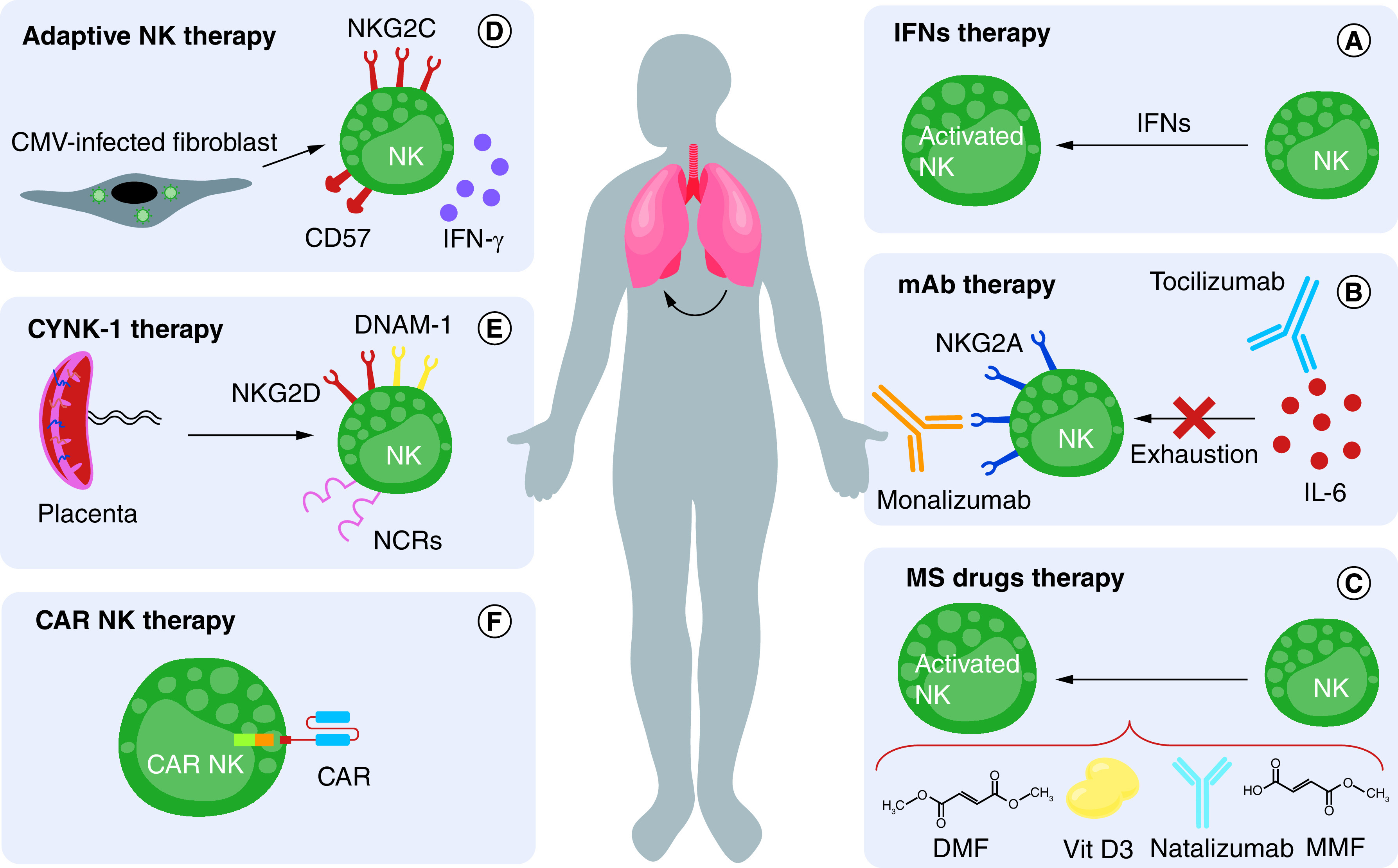
Suggesting NK cell-based therapies for COVID-19. **(A)** The use of IFNs can prevent the spread of infection by activating NK cells and preventing adjacent cells from being infected. They do this by increasing the expression of antiviral genes, and this inhibits the accumulation of neutrophils. **(B)** The use of an IL-6 inhibitor called tocilizumab can prevent NK cell exhaustion. In addition, the inhibition of NKG2A may be effective in preventing pathological effects of NK cells. **(C)** Multiple sclerosis medications including vitamin D3, MMF and natalizumab, may be used to counteract the exhaustion of NK cells by SARS-CoV-2. **(D)** Adaptive NK cells, a group of NK cells that express higher levels of NKG2C/CD57 and secrete more IFNs, can be produced in the laboratory using CMV-infected fibroblasts. These can be considered a treatment option for COVID-19 patients. **(E)** CYNK-001 is a group of NK cells derived from umbilical cord stem cells. They can detect virus infected cells and eradicate infection using stimulatory receptors such as NKG2D, DNAM and NCRs. **(F)** Genetically engineered cells called CAR-NK cells can also be used to treat COVID-19. DMF: Dimethyl fumarate; MMF: Monomethyl fumarate; NK: Natural killer cell; CAR: Chimeric antigen receptors; IFN: interferons; CMV: Cytomegalovirus; MS: Multiple sclerosis; mAb: Monoclonal antibody; DNAM-1: DNAX accessory molecule-1; NCRs: Natural cytotoxicity receptors.

As mentioned, IL-6 plays an important role in the exhaustion and dysfunction of NK cells in patients with COVID-19 [101], so the use of an IL-6 inhibitor called tocilizumab can prevent NK cell exhaustion (Figure 2) [101,102]. Clinical trials are underway to evaluate the effects of this inhibitor in patients (NCT04335071), as IL-6 can increase NKG2A and CD39 expression. This is important in preventing the response of NK cells to virus infected cells and preventing the immunomodulatory function of NK cells [24]. The use of tocilizumab in the treatment of cytokine storms by chimeric antigen receptor (CAR) T cells has been effective, which indicates the importance of this inhibitor in keeping NK cells active and in neutralizing the cytokine storm caused by SARS-CoV-2 [102,103]. Inhibitors of TNF-α, another inflammatory cytokine that plays an important role in the severity of the disease, have also been suggested as a therapeutic approach for COVID-19 [104]. Since TNF-α is the by-product of NK cells and plays a role in their effector function, it seems that the use of TNF blockers may adversely effect NK cells. However, this approach will require additional future study because studies have demonstrated that COVID-19 patients receiving TNF blockers showed a lower probability of hospitalization [105].

Monalizumab, the inhibitor of the NKG2A receptor, has been used to treat different types of malignancies including leukemia. Due to the important role of NKG2A in the exhaustion of NK cells, its inhibition may be effective in preventing pathological effects of the immune system (Figure 2) [106]. Detailed studies should be conducted into the use of this medication in COVID-19 patients, since NKG2A is expressed on the surface of other immune cells such as T cells and thus its inhibition may exacerbate the disease [107].

One of the most interesting ways to increase NK cell activity may be to use multiple sclerosis medications. These include vitamin D3, dimethyl fumarate, monomethyl fumarate and natalizumab, which may be used to counteract the exhaustion of NK cells by SARS-CoV-2 (Figure 2). Vitamin D supplementation has beneficial impacts on immune function, particularly in the context of autoimmunity. Vassiliou *et al.* have shown that there is a significant relationship between the numbers of NK cells in vitamin D deficient and insufficient COVID-19 patients [108]. More research is needed on the use of these medications [109,110].

IFNs play an essential role in preventing the spread of viral agents to among host cells and in activating immune system cells to fight the virus. Like Middle East respiratory syndrome and SARS, SARS-CoV-2 both reduces the production of these cytokines and disrupts their signaling pathway [47]. The use of IFNs therefore seems reasonable in patients with COVID-19 as a way to activate NK cells promptly and prevent adjacent cells from being infected via an increased expression of antiviral genes. They may also inhibit the accumulation of neutrophils, which are a major cause of lung tissue damage (Figure 2) [111]. However, it should be noted that the timing of the use of these cytokines is very important. IFNs should preferably be administered in the early stages, as their use in the final stages of disease can increase the immunopathogenesis [47,112].

CYNK-001 is a group of NK cells derived from umbilical cord stem cells that has a remarkable ability to fight viral infections, and thus they have been used to treat malignancies such as multiple myeloma and acute myeloid leukemia. This group of laboratory produced NK cells has the ability to detect virus infected cells and eradicate infection using stimulatory receptors such as NKG2D, DNAM and NCRs (Figure 2) [48,113]. Clinical trials using these cells for patients with COVID-19 are currently ongoing (NCT04365101). When using these types of cells, the timing must be considered, as their use in the early stages of the disease will likely result in better outcomes.

CAR cells are a promising treatment to treat cancers and various viral diseases, and even autoimmune diseases (Figure 2). In these cells, receptor(s) that are generally not present on the cell surface are genetically engineered to present on the cell surface. One of the most important CAR cells is a CAR-T cell, engineered T cells that are highly effective in the treatment of cancer, especially blood cancers. However, there are several challenges to the use of CAR-T cells, including the high cost and risks of cytokine storms [114]. Researchers have begun to produce genetically engineered CAR-NK cells, a less expensive alternative that is less likely to cause cytokine storm. These cells are being used to treat cancers and viral diseases in clinical trials [61,115], and a clinical trial is currently underway to test the ability of CAR-NK cells that express the ACE2 receptor on their surface to treat COVID-19 patients (NCT04324996). If this clinical trial is successful, these could be used to treat patients with COVID-19. Recently, a CAR-NK cell therapy that secrets IL-15 to improve cell survival and expresses CAR with an extracellular ACE2 domain to target the SARS-CoV-2 Spike protein has demonstrated promising effects against VSV-SARS-CoV-2 chimeric viral particles and the recombinant SARS-CoV-2 Spike protein subunit S1. These CAR-NK cells by enhanced cytotoxicity as well as TNF-α and IFN-γ production [116].

Adaptive NK cell refers to a group of NK cells with memory properties, such as longevity, cytotoxicity and higher cytokine secretion. These cells are usually developed in response to the CMV virus and resist reinfection. This group of NK cells generally express the NKG2C receptor, secrete more IFNs and express the maturation marker (CD57) at a higher level than in normal NK cells [117]. These cells also have roles in hematopoietic stem cell transplantation and the treatment of leukemia [118]. Studies have shown that the severity of COVID-19 in kidney transplant recipients infected with both SARS-CoV-2 and CMV is less than in recipients with COVID-19 without CMV infection [119]. This indicates the effect of adaptive NK cells on COVID-19. In addition to being naturally developed in the body of people infected with CMV, these cells can be produced in the laboratory using CMV-infected fibroblasts and can be considered a treatment option for COVID-19 patients (Figure 2) [120]. A phenotype investigation study also showed the presence of memory like CD57^+^ NKG2C^+^ NK cells in the peripheral blood of COVID-19 patients. This indicates the promising potential of convalescent therapy to treat SARS-CoV-2 infection, due to the unique cytotoxic activity of this subtype against different viral infections [66].

With their C5 convertase enzyme function, alveolar neutrophils and macrophages can form C5a by cleavage of the complement molecule C5. The inflammatory mediator functions of C5a are activated upon binding C5aR on different immune cells, including NK and NK T cells. This induces the production of different cytokines and chemokines in various cells, reducing the role of NK and NK T cells in some inflammatory situations like sepsis. Due to the high similarities of sepsis and SARS-CoV-2, and the presence of increased C5a serum levels in COVID-19 patients, it has been assumed that this inflammatory mediator might play a pivotal role in cytokine storms during COVID-19. Based on this, a clinical trial (NCT04371367) has started to evaluate the hyperinflammatory responses in patients diagnosed with SARS-CoV-2, using avdoralimab [121–125]. The summary of clinical trials based on NK cells is provided in Table 2 according to https://clinicaltrials.gov/.

**Table 2.** A summary of NK-cell-based clinical trials.

No.	Study title	Trial no.	Interventions	Location	Status
1	A phase I/II study of universal off-the-shelf NKG2S-ACE2 CAR NK cells for therapy of COVID-19	NCT04324996	NK cells, IL15-NK cells, NKG2D CAR-NK cells, ACE2 CAR-NK cells, NKG2D-ACE2 CAR-NK cells	China	Recruiting
2	Monocyte and NK cells activity in COVID-19 patients	NCT04375176	Diagnostic test: study of immune-mediated mechanisms in patients tested positive for SARS-CoV-2 Phenotypic and functional analysis of monocytes and NK cells	Italy	Recruiting
3	Off-the-shelf NK cells (KDS-1000) as immunotherapy for COVID-19	NCT04797975	KDS-1000	Not yet been determined	Not yet recruiting
4	Phase I clinical trial on NK cells for COVID-19	NCT04634370	NK Cells infusion	Brazil	Not yet recruiting
5	NK cells treatment for COVID-19	NCT04280224	NK Cells	China	Recruiting
6	Immune cell subgroups in COVID-19 patients	NCT04531319	Flow cytometric analysis	Turkey	Completed
7	NK cell (CYNK-001) infusion in adults with COVID-19	NCT04365101	CYNK-001	USA	Recruiting
8	Phase I/II clinical study of immunotherapy based on adoptive cell transfer as a therapeutic alternative for patients with COVID-19 in Colombia	NCT04344548	Allogenic NK cell transfer	Colombia	Not yet recruiting

NK: Natural killer cell.

## Conclusion

This paper discussed the effects of SARS-CoV-2 on NK cells during infection, the effect of vaccination on NK cells and possible therapies based on NK cell functions. During SARS-CoV-2 infection, the virus hampers the proper immune response by impairing the functionality of NK cells. This leads to an improper immune response in the initial stages and can cause cytokine storms in the later stages [4]. The virus can also cause the exhaustion of NK cells by increasing expression of the inhibitory receptor NKG2A, and hindering proper production and signaling of IFNs [25,126]. The exhaustion of NK cells, as well as the reduction of activating receptors such as NKG2D, means that they are unable to control hyperactive immune cells in the later stages of the infection and thus result in severe damage to tissues, especially lung tissue [70]. Reduction of the number of NK cells in the peripheral blood is another technique employed by SARS-CoV-2 to evade clearance [17].

Limited studies have evaluated the effect of COVID-19 vaccines on NK cells. It has been shown that DNA vaccines and BNT162b2 can affect NK cell response and may influence the efficacy of the vaccine [95,96]. Therefore, future studies are needed to assess the effect of different COVID-19 vaccines and their components on NK cells.

We have also discussed NK cell-based treatments for COVID-19. Based on previous information, the use of anti-NKG2A (Monalizumab) and anti-IL-6 (tocilizumab) antibodies may be useful in controlling the disease, as well as restoring the function of NK cells [102,106]. It has been shown that the use of therapeutic drugs traditionally given to MS patients can play an important role in increasing NK cells activity [109]. The use of IFNs at the beginning stages of the disease may also prevent the spread of the virus [111]. Cellular therapies such as CYNK-001, CAR-NK and adaptive NK cells are also promising for use to treat COVID-19 patients.

## Future perspective

For a better understanding of the role of NK cells in SARS-CoV-2, we need further strategic research. This should focus on the changes in the expression of NK cell receptors such as NKG2A, NKG2D and KIRs family, the exhaustion of NK cells and the education process. The effects of SARS-CoV-2 vaccines on NK cells are also unclear. Based on limited reports, it seems that vaccination can affect NK cell function and phenotype. Various studies can be performed to investigate the effect of different vaccine platforms as well as adjuvants on the proliferation, phenotype or function of NK cells. Finally, it seems that NK cell-based therapy and NK cell targeting therapy could be useful strategies for controlling the disease. Clinical trials testing the use of these cells for treating patients with COVID-19 are currently ongoing, and may be a new target for effective treatment.

**Executive summary**

- In this article, we reviewed the effect of SARS-CoV-2 on the count and function of natural killer (NK) cells during infection, the effect of vaccination on NK cells and also some NK cell-based therapies which can help to treat the disease.

**SARS-CoV-2 affects the frequency, phenotype & function of the NK cells**

- In SARS-CoV-2 infection the number of NK cells is decreased, which is directly correlated to the severity of the disease.
- SARS-CoV-2 interferes with the production and signaling of IFNs and prevents the activation of NK cells.
- SARS-COV-2 causes exhaustion of NK cells by increasing the expression of inhibitory receptors.
- SARS-CoV-2 can affect the education process of the NK Cells.

**Induction of effective immune response by COVID-19 vaccines with involvement of NK cells**

- Vaccination has been shown to induce anti-Spike dependent NK cell responses in vaccinated rhesus macaques.
- Vaccination of haemato-oncological patients with BNT162b2 demonstrated that the lack of serological response is associated with low levels of NK cells.
- A positive correlation has been seen between the frequency of NKG2C+ NK cells and anti-SARS-CoV-2 Ab titers following BNT162b2 mRNA vaccination.

**NK cell-based & NK cell-targeting therapies for COVID-19**

- The use of an IL-6 inhibitor called tocilizumab can prevent NK cell exhaustion.
- The use of IFNs can prevent the spread of infection by activating NK cells, and prevent adjacent cells from being infected through increasing the expression of antiviral genes.
- The inhibition of NKG2A may be effective in preventing pathological effects of the NK cells.
- Multiple sclerosis medications include vitamin D3, dimethyl fumarate, monomethyl fumarate and natalizumab, may be used to counteract the exhaustion of NK cells by SARS-CoV-2.
- Adaptive NK cells, a group of NK cells that express higher NKG2C, CD57 and secrete more IFNs, can be produced in the laboratory using CMV-infected fibroblasts and can be considered a treatment option for COVID-19 patients.
- CYNK-001 and CAR NK cells could also be used to treat COVID-19.

**Conclusion**

- In SARS-CoV-2 infection, the virus hampers proper immune response by impairing NK cell functionality, leading to an improper response in the initial stages and cytokine storms in the later stages.
- The virus causes exhaustion of NK cells by increasing NKG2A expression and hindering proper production and signaling of IFNs.
- It was shown that DNA vaccines and BNT162b2 can affect NK cell response.
- The use of anti-NKG2A and anti-IL-6 may be useful in controlling the disease.
- The use of therapeutic drugs for MS patients can increase NK cells activity.
- CYNK-001, CAR-NK and adaptive NK cells could be used to treat COVID-19 patients.

**Future perspective**

- For better understanding of the role of NK cells in SARS-CoV-2,, we need further strategic research with the focus on NK cell activation, exhaustion and education.
- Various studies can be performed to investigate the effects of different vaccine platforms as well as adjuvants, on NK cells.
- Finally, NK cell-based therapy can be a useful strategy for controlling the disease and may be new targets for effective treatment.
